# Radiomics in Gastric Cancer: First Clinical Investigation to Predict Lymph Vascular Invasion and Survival Outcome Using ^18^F-FDG PET/CT Images

**DOI:** 10.3389/fonc.2022.836098

**Published:** 2022-03-30

**Authors:** Liping Yang, Wenjie Chu, Mengyue Li, Panpan Xu, Menglu Wang, Mengye Peng, Kezheng Wang, Lingbo Zhang

**Affiliations:** ^1^ Department of PET-CT, Harbin Medical University Cancer Hospital, Harbin, China; ^2^ College of Bioinformatics Science and Technology, Harbin Medical University, Harbin, China; ^3^ Oral Department, The Second Affiliated Hospital of Harbin Medical University, Harbin, China

**Keywords:** gastric cancer, PET-CT, nomogram, lymph vascular invasion, survival prognosis, radiomics

## Abstract

**Background:**

Lymph vascular invasion (LVI) is an unfavorable prognostic indicator in gastric cancer (GC). However, there are no reliable clinical techniques for preoperative predictions of LVI. The aim of this study was to develop and validate PET/CT-based radiomics signatures for predicting LVI of GC preoperatively. Radiomics nomograms were also established to predict patient survival outcomes.

**Methods:**

This retrospective study registered 148 GC patients with histopathological confirmation for LVI status, who underwent pre-operative PET/CT scans (Discovery VCT 64 PET/CT system) from December 2014 to June 2019. Clinic-pathological factors (age, gender, and tumor grade, etc.) and metabolic PET data (maximum and mean standardized uptake value, total lesion glycolysis and metabolic tumor volume) were analyzed to identify independent LVI predictors. The dataset was randomly assigned to either the training set or test set in a 7:3 ratios. Three-dimensional (3D) radiomics features were extracted from each PET- and CT-volume of interests (VOI) singularly, and then a radiomics signature (RS) associated with LVI status is built by feature selection. Four models with different modalities (PET-RS: only PET radiomics features; CT-RS: only CT radiomics features; PET/CT-RS: both PET and CT radiomics features; PET/CT-RS plus clinical data) were developed to predict LVI. Patients were postoperatively followed up with PET/CT every 6-12 months for the first two years and then annually up to five years after surgery. The PET/CT radiomics score (Rad-scores) was calculated to assess survival outcome, and corresponding nomograms with radiomics (NWR) or without radiomics (NWOR) were established.

**Results:**

Tumor grade and maximum standardized uptake value (SUVmax) were the independent LVI predictor. 1037 CT and PET 3D radiomics features were extracted separately and reduced to 4 and 5 features to build CT-RS and PET-RS, respectively. PET/CT-RS and PET/CT-RS plus clinical data (tumor grade and SUVmax) were also developed. The ROC analysis demonstrated clinical usefulness of PET/CT-RS plus clinical data (AUC values for training and validation, respectively 0.936 and 0.914) and PET/CT-RS (AUC values for training and validation, respectively 0.881 and 0.854), which both are superior to CT-RS (0.838 and 0.824) and PET-RS (0.821 and 0.812). SUVmax and LVI were independent prognostic indicators of both OS and PFS. Decision curve analysis (DCA) demonstrated NWR outperformed NWOR and was established to assess survival outcomes. For estimation of OS and PFS, the C-indexes of the NWR were 0. 88 and 0.88 in the training set, respectively, while the C-indexes of the NWOR were 0. 82 and 0.85 in the training set, respectively.

**Conclusions:**

The PET/CT-based radiomics analysis might serve as a non-invasive approach to predict LVI status in GC patients and provide effective predictors of patient survival outcomes.

## Introduction

Gastric cancer (GC) is currently one of the most common malignant tumors, accounting for the second-highest number of cancer-related fatalities worldwide, seriously threatening human health and life safety (1). Furthermore, approximately 70% of cases occur in Asia, with China accounting for at least half of all cases. Surgical resection is taken as the standard treatment approach for GC that is surgically resectable (2). Unfortunately, the poor survival prognosis arising from postoperative tumor recurrence is still a clinical dilemma. It has been reported that the recurrence rate of GC patients within two years after radical resection was 61.7%, and the average recurrence time was 24.3 months. Especially, 90% of patients with stage III GC had a recurrence rate of 50% and 40% in the first and second years after surgery (3, 4). At present, there are currently no efficient and reliable prognostic bio-markers for identifying high-risk groups for adjuvant therapy in clinical practice.

Malignant tumor cell metastasis is the leading cause of death in patients with malignant tumors, in which lymphatic metastasis is the main way. Lymph vascular invasion (LVI) refers to the infiltration of tumor cells in the lumen of arteries, veins, or lymphatic vessels during histologic examination with hematoxylin and eosin (H&E) stains, D2-40 and CD31 stains, which has previously been demonstrated to prompt the local recurrence and distant metastasis of tumors (5, 6). For instance, LVI has been reported to be an independent prognostic factor for the overall survival (OS) and disease-free survival (PFS) of breast cancer patients (7, 8). Thus, accurate identification of LVI status is conductive to develop personalized treatment planning for breast cancer patients. Meanwhile, a series of studies have found that the occurrence of pathological LVI was closely associated with the progression of GC and poor clinical prognosis. The incidence of LVI was 25% and 44% in moderately and well differentiated and poorly differentiated gastric cancers, respectively, while the 5-year survival rate of GC was only 37.7% in patients with LVI-positive, which was significantly lower than 59.9% of patients with LVI-negative (9–12). Although LVI is considered to be a key prognostic factor of unsatisfactory survival outcomes in various cancers, accurate identification of LVI status prior to operation is still difficult because LVI is mainly found through postoperative pathology.

^18^F-fluorodeoxyglucose(^18^F-FDG) positron emission tomography-computed tomography (PET-CT), as a prospect imaging modality, plays a vital role in preoperative staging, treatment efficacy evaluation, tumor residual, and recurrence identification of GC. Nevertheless, predicting LVI of GC patients using quantitative PET metabolic parameters has received minimal attention. Lin et al. found that ratio maximum standardized uptake values (SUVmax) to mean standardized uptake values (SUVmean) is an independent predictor of LVI in hepatocellular carcinoma (13). Noda et al. reported that SUVmax of lung cancer could be employed for the identification of LVI (14). Unfortunately, the clinical usefulness of all these metabolic parameters in predicting LVI has not been demonstrated in GC, which needs to be deeply investigated.

Radiomics, which transformed digital medical images into high-throughput data, is a promising and non-invasive method that can extract high-throughput features (such as shape, intensity, and texture features) (15). It captures relationships between image voxels that may not be perceived by the naked eyes of physicians-even experienced radiologists, which can contribute to the diagnostic and predictive accuracy of the disease (16). Xu et al. reported that total lesion glycolysis (TLG) might be the best indicator for predicting lymph vascular space invasion (LVSI) in cervical cancer without lymphatic metastasis (17). Nie et al. investigated the clinical value of the PET/CT-based radiomics analysis in predicting LVI status, and the results demonstrated the favorable predictive efficacy for LVI status in lung adenocarcinoma patients (18). Several works focused on predicting the LVI status of GC using computed tomography (CT)-derived radiomics features have previously been reported. Chen et al. demonstrated that radiomics analysis based on contrast-enhanced computed tomography (CECT) may help to predict LVI status and PFS (19). In Meng et al.’s study, models constructed with two-dimensional (2D) radiomics features revealed comparable performances with those constructed with three-dimensional (3D) features in predicting LVI status (20). However, to our knowledge, no previous study has focused on the clinical value of PET-based radiomic signatures in the preoperative prediction of LVI in GC.

In the present study, we intended to develop and validate the PET/CT-based radiomics models for preoperatively predicting the LVI status of GC. Furthermore, we also investigated whether the PET/CT-based nomogram can be applied as a non-invasive method to assess patient survival outcomes.

## Materials and Methods

Ethical approval was obtained for this retrospective study, and the need for written informed consent was waived. The enrolment flowchart of this study is displayed in **Supplementary Figure 1**. A total of 148 patients with pathologically confirmed GC from December 2014 to June 2019 were enrolled in this study according to the following inclusion criteria:1) PET/CT scans were performed before surgery;2) GC patients with clear pathologically confirmed LVI on surgical resection specimens;3) No previous anti-tumor therapy before surgery such as radiotherapy, chemotherapy and neoadjuvant therapy;4) Patients with detailed clinical data and follow-up information (OS and PFS were followed up until September 30, 2020). The exclusion criteria were as follows: 1) Poor image quality (artifacts related to patient motion, which was assessed by a senior radiologist who has 15-year specialized experience); 2) History of other malignant tumors. Clinical information was obtained through clinical medical record retrieval, including age, gender, lymph node metastasis, cTNM, T stage, N stage, M stage, molecular subtype, tumor grade, tumor thickness, carcinoembryonic antigen (CEA), carbohydrate antigen 125 (CA125), carbohydrate antigen199 (CA199), SUVmax, SUVmean, metabolic tumor volume (MTV) and TLG.

### Image Acquisition

Prior to scanning, all patients were required to fast for at least 6 hours. All patients’ blood glucose levels should be kept below 11.0 mmol/L. PET/CT images were acquired using the Discovery VCT 64 PET/CT system (GE Healthcare, Milwaukee, USA). A total of 1000-1200ml contrast agent (Meglumine diatrizoate at a concentration of 2%) was injected into the patients 15 minutes before the examinations to fill the gastric cavity, which is a cheap, effective and well-tolerated intracavitary contrast agent with minimal adverse effects. A 3.78 MBq/kg dose of ^18^F-FDG was administered intravenously, and approximately one hour later, whole-body CT scanning was performed. Specific imaging parameters were listed as follows: tube voltage 140 kV, tube current 140 mA, slice thickness 3 mm, reconstruction interval 3 mm, matrix size 512 × 512, and field of view 650 mm. After the CT scan, the emission scan was followed by a 1.5-2 min transmission scan per bed position. After the completion of the CT scan, the PET emission scan was followed by a 2 min per bed position. Image reconstructions were performed based on the 3D ordered subset expectation-maximization algorithm (2 iterations and 17 subsets).

### Image Analysis

The PET/CT images were analyzed by two radiologists blinded to the clinical and pathological results, (Reader 1, P.X and Reader 2, C.G with 10- and 15-years’ experience in the interpretation of PET/CT images, respectively). The metabolic parameters were measured by drawing a region-of-interest (ROI) on the axial PET image based on a threshold of 40% of SUVmax using commercial software (PET VCAR; GE Healthcare, USA). Any disagreement was resolved by consensus. SUVmax was defined at the highest value on one pixel with the highest counts within the ROI (21).

### Tumor Segmentation and Radiomics Feature Extraction

**Figure 1:**
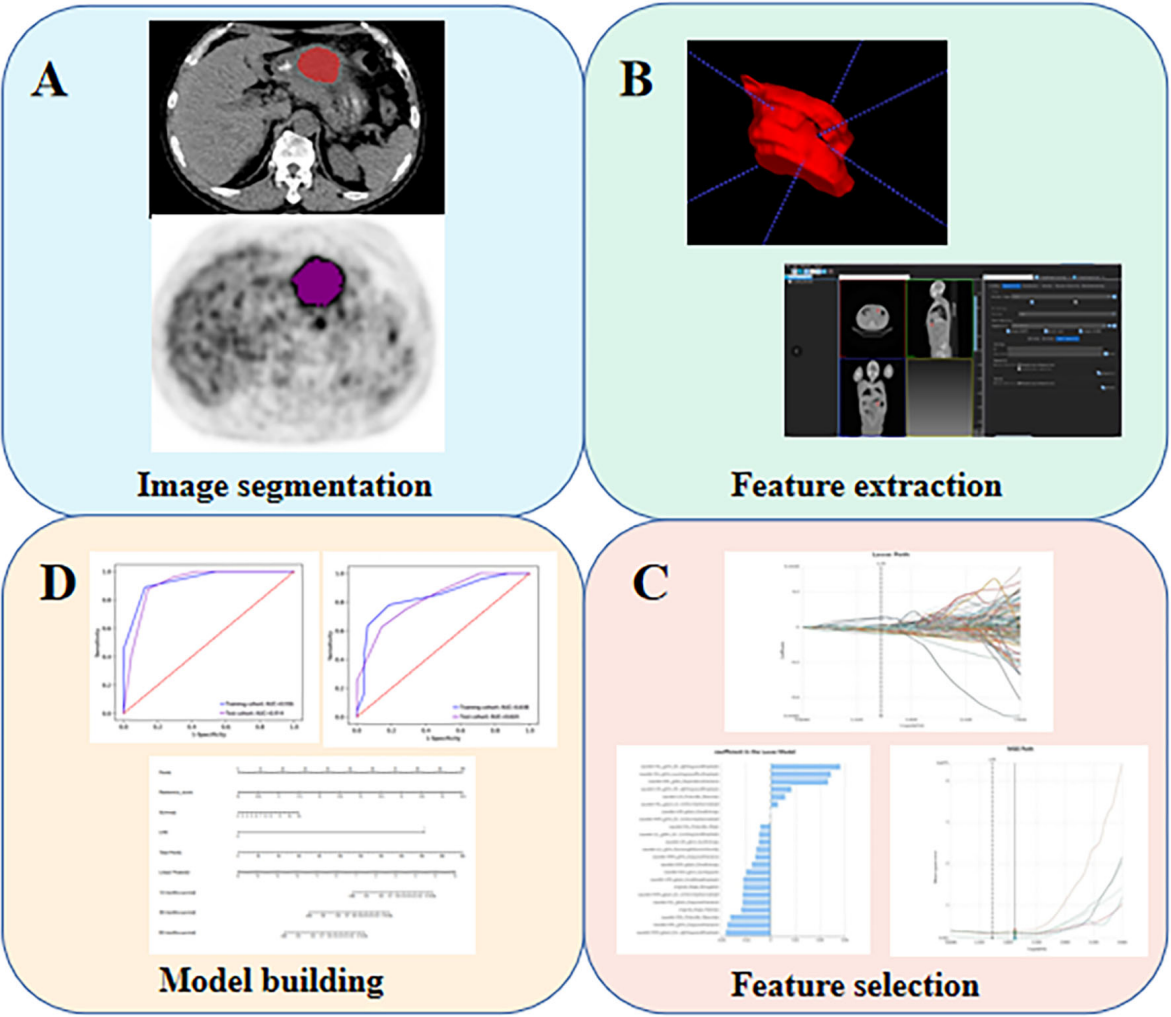
The flow diagram of this study. **(A)** Image segmentation; **(B)** Feature extraction; **(C)** Feature selection; **(D)** Model building.

The overview of the radiomics workflow is displayed in **Figure 1**. Axial PET and CT Digital Imaging and Communications in Medicine images obtained from the Picture Archiving and Communication System were applied for tumor segmentation. The tumor lesion was delineated on axial PET and CT images using LIFEx software (open-source software; www.lifexsoft.org/index.php) (**Figure 1A**). All 3D segmentation was first delineated automatically by means of a fixed threshold of 40% of the SUVmax, which were corrected by a radiologist manually afterward, blinded to surgical and pathological results.

We adopted three steps to preprocess the PET and CT images prior to feature extraction (22). Firstly, we resampled all images to a uniform voxel size of 1 mm × 1 mm × 1 mm using linear interpolation to minimize the influence of different layer thicknesses. Secondly, based on the gray-scale discretization process (bin width for CT = 25, bin width for PET = 0.1), we convert the continuous image into discrete values. Finally, we use the Laplacian of Gaussian and wavelet image filters to eliminate the mixed noise in the image digitization process in order to obtain low- or high-frequency features. Radiomics features were extracted from each PET-derived volume of interest (VOI) and CT-derived VOI by applying dedicated AK software (Artificial Intelligence Kit; GE Healthcare), which is in compliance with image biomarker standardization initiative guidelines (23). A total of 2074 radiomics features were extracted from each VOIs (1037 for CT, 1037 for PET) including (i) 198 for first-order feature, (ii) 14 for shape feature, (iii) 264 for gray level co-occurrence matrix (GLCM) feature, (iv) 176 for gray level size zone matrix (GLSZM) feature, (v) 176 for gray level run length matrix (GLRLM) feature, (vi) 55 for neighborhood gray tone difference matrix (NGTDM) feature, (vii) 154 for gray level dependence matrix (GLDM) feature.

### Radiomic Feature Selection and Model Development

After the radiomics features extraction, Z-score normalization was done on each radiomics feature. In addition, the same preprocessing procedure was also applied to the testing set. The dataset was randomly assigned to either the training set or test set in 7:3 ratios. All cases in the training set were used to train the predictive model, while cases in the test set were utilized to independently evaluate the model’s performance.

Firstly, intra- and inter-class correlation coefficients (ICCs) were calculated to assess the intra- and inter-observer reproducibility. Reader 1 and Reader 2 drew the VOIs of 40 cases (20 LVI-present GCs and 20 LVI-absent GCs) of CT images and PET images randomly selected from the whole cohort. Reader 1 repeated the segmentations two weeks later. ICC greater than 0.80 indicated good agreement of feature extraction. The VOI segmentation for the remaining cases were performed by Reader 1. Next, the feature selection was carried out by using a step-by-step selection method. Firstly, univariate logistic regression analysis with the Mann-Whitney U test was utilized to select features with *P*-value< 0.05 for the subsequent analysis. Secondly, multivariate logistic regression analysis was applied to choose features closely related to LVI status. Finally, a subset of the most informative features was retained using the least absolute shrinkage and selection operator (LASSO) method. The k-Nearest Neighbor (KNN) was applied for model construction, and four sets of machine learning models (a CT-RS, a PET-RS, a PET/CT-RS, a PET/CT-RS incorporating clinical and metabolic parameters) were developed to predict LVI of GC. The diagnostic performance of the radiomics models was evaluated regarding the area under the curve (AUC), sensitivity, specificity, and accuracy.

### Construction of Radiomics Nomograms

In this study, among all pathologic and therapeutic factors, SUVmax and pathologic LVI were demonstrated to be associated with survival prognosis, which were incorporated into the nomogram’s construction (**Supplementary Tables 4–7**). A PET/CT radiomics score (Rad-scores) was calculated, and corresponding nomograms with radiomics (NWR) or without radiomics (NWOR) were established by incorporating the independent LVI predictors as well as the Rad-score to assess survival outcome. Calibration curve analysis and Decision curve analysis (DCA) were performed to assess the clinical value of the nomograms.

### Follow Up and Survival Analysis

Patients were postoperatively followed up every 6-12 months for the first 2 years and then annually up to five years. The endpoints of this study were PFS and OS. PFS is defined as the time interval from surgery to the recurrence or progression of the disease. OS is defined as the time interval from surgery to death. Survival curves were drawn using the Kaplan-Meier approach and compared using the log-rank test. All the prognostic factors (including pathologic LVI status, gender, age, lymph node metastasis, tumor grade, molecular subtype, T stage, N stage, M stage, cTNM, CEA, CA125, CA199, Tumor thickness, SUVmax, SUVmean, MTV and TLG were evaluated by univariate analysis using the Kaplan-Meier approach. Statistically significant variables were analyzed for the multivariate Cox forward stepwise regression model to select independent predictors of OS and PFS.

### Statistical Analyses

Univariate analysis (chi-square test or Mann-Whitney U test) and multivariate logistic regression was used to screen out final significant variables by using SPSS software (Version 25.0, IBM). ICC, receiver operating curve (ROC) analysis, calibration plots, DCA, and survival analysis were performed with R statistical software (version 3.5.1). A two-sided *P*-value< 0.001 was used as the criterion to indicate a statistically significant difference.

## Results

### Patient Characteristics

A total of 148 patients (103 males and 45 females; average age 61; median age 60 years; age range 35-85 years) were recruited for this study, including 69 cases of LVI-present and 79 cases of LVI-absent. The clinic-pathological variables and PET metabolic parameters of all patients are displayed in **Table 1**. In univariate logistic regression analysis, there was no significant statistical difference in gender, age, molecular subtype, T stage, M stage, cTNM, CEA, CA125, CA199, tumor thickness, SUVmean and MTV between LVI-present and LVI-absent groups (*P* > 0.001), while lymph node metastasis, tumor grade, N stage, SUVmax and TLG were statistically significant (*P* < 0.001). Among these parameters, tumor grade and SUVmax were further shown to be independent LVI predictors (**Supplementary Table 2**).

**Table 1:** Baseline clinical characteristics of patients.

Clinical factors	LVI-absent	LVI-present	X²/Z	*P*
**Gender**			0.3610	0.5479
Female	19 (27.9)	26 (32.5)		
Male	49 (72.1)	54 (67.5)		
**Lymph node metastasis**			23.1482	< 0.01
Negative	33 (48.5)	10 (12.5)		
Positive	35 (51.5)	70 (87.5)		
**Tumor grade**			35.6672	< 0.01
Well differentiated	7 (10.3)	1 (1.25)		
Middle differentiated	43 (63.2)	19 (23.8)		
Poorly differentiated	18 (26.5)	60 (75.0)		
**Molecular subtype**			4.2472	0.2360
Undifferentiated	11 (16.2)	24 (30.0)		
Diffuse type	21 (30.9)	23 (28.8)		
Mixed type	18 (26.5)	18 (22.5)		
Intestinal type	18 (26.5)	15 (18.8)		
**T stage**			6.4222	0.0928
T1	17 (25.4)	13 (16.3)		
T2	36 (53.7)	40 (50.0)		
T3	14 (20.9)	22 (27.5)		
T4	0 (0.0)	5 (6.25)		
**N stage**			85.4190	< 0.01
N0	33 (48.5)	6 (7.5)		
N1	29 (42.6)	6 (7.5)		
N2	4 (5.9)	38 (47.5)		
N3	2 (2.94)	30 (37.5)		
**M stage**			3.1613	0.0754
M0	38 (55.9)	56 (70.0)		
M1	30 (44.1)	24 (30.0)		
**cTNM**			6.3146	0.0973
I	24 (35.3)	18 (22.5)		
II	11 (16.2)	7 (8.8)		
III	3 (4.4)	5 (6.3)		
IV	30 (44.1)	50 (62.5)		
**Age**	62.43 ± 9.64	61.33 ± 10.35	0.67	0.5067
**CEA**	2.19 ± 3.07	20.67 ± 78.25	-1.93	0.0554
**CA125**	14.34 ± 43.34	20.53 ± 47.75	-0.82	0.4136
**CA199**	63.12 ± 65.73	126.62 ± 309.72	-1.65	0.1019
**SUVmax**	6.31 ± 2.25	9.20 ± 2.87	-6.71	< 0.01
**Tumor thickness**	1.61 ± 0.59	1.70 ± 0.56	-0.90	0.3701
**TLG**	65.63 ± 62.55	99.04 ± 55.19	-3.43	< 0.01
**SUVmean**	8.35 ± 4.97	9.32 ± 3.93	-1.32	0.1877
**MTV**	9.31 ± 5.72	8.99 ± 4.67	0.38	0.7037

SUVmax, maximum standardized uptake value; SUV, mean mean standardized uptake value; TLG, total lesion glycolysis; MTV, metabolic tumor volume; CEA, carcinoembryonic antigen; CA125, carbohydrate antigen 125; CA199, Carbohydrate antigen199; LVI, lymph vascular invasion.

### Intra and Inter-Observer Reproducibility of Feature Extraction

The intra-observer ICC ranged from 0.811 to 0.920, and inter-observer ICCs were ranged from 0.740 to 0.902. Therefore, a favorable intra- and inter-observer reproducibility of radiomics feature extraction was observed in our study.

### Performance of the Four Models

**Figure 2:**
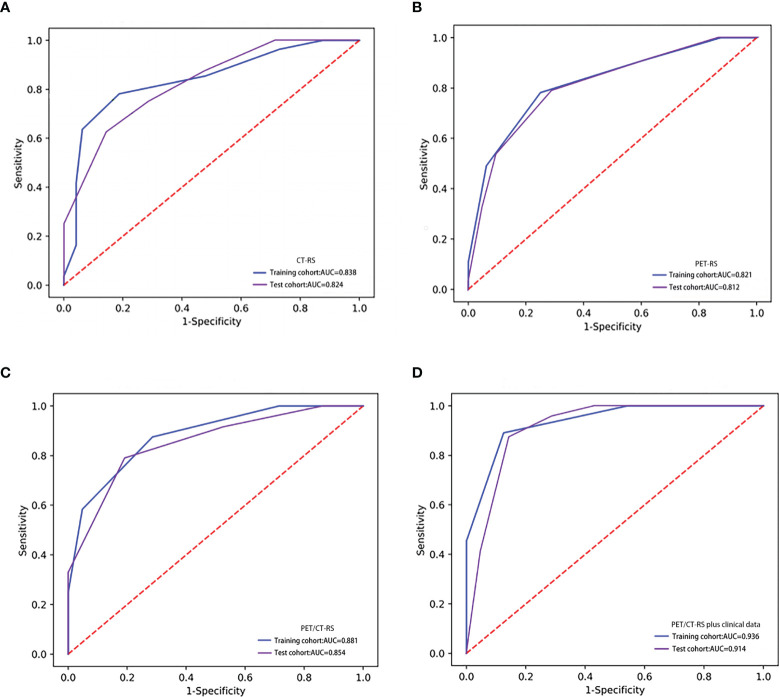
ROCs of different radiomics models in the training and test set. **(A)** The ROC of CT-RS; **(B)** The ROC of PET-RS; **(C)** The ROC of PET/CT-RS; **(D)** The ROC of PET/CT-RS incorporating clinical and metabolic parameters.

After proper feature selection, 4, 5, 9, and 11 RSs were selected respectively to develop the CT-RS, PET-RS, PET/CT-RS, and clinical parameters integrated models for predicting LVI status in GC. After using a step-by-step selection method, four CT and five PET radiomics features were eventually selected to build CT-RS and PET-RS, respectively. Radiomics features and corresponding coefficients and their significance are listed in **Supplementary Table 3**. The ROC analysis demonstrated the clinical usefulness of the integrated model and PET/CT-RS, which both are superior to the CT-RS and PET-RS. All results regarding diagnostic efficacy were demonstrated in **Table 2**, and the ROC curves were as displayed in **Figure 2**. The ROC analysis demonstrated a favorable clinical usefulness of PET/CT-RS plus clinical data (AUC values for training and validation, respectively 0.936 and 0.914) and PET/CT-RS (AUC values for training and validation, respectively 0.881 and 0.854), which both are superior to CT-RS (0.838 and 0.824, both *P* values < 0.001) and PET-RS (0.821 and 0.812, both *P* values < 0.001). The accuracy, precision, sensitivity and specificity were 0.796, 0.827, 0.782 and 0.812 for CT-RS model; 0.767, 0.782, 0.782 and 0.75 for PET-RS model; 0.806, 0.857, 0.764 and 0.854 for PET/CT-RS; 0.883, 0.891, 0.891 and 0.875 for PET/CT-RS incorporating clinical and metabolic parameters, respectively.

**Table 2:** Diagnostic Performance of different radiomics models.

	CT-RS		PET-RS		PET/CT-RS		PET/CT-RS incorporating clinical and metabolic parameters	
	Training set	Test set	Training set	Test set	Training set	Test set	Training set	Test set
**Accuracy**	0.796	0.733	0.767	0.756	0.806	0.800	0.883	0.867
**Precision**	0.827	0.750	0.782	0.760	0.857	0.826	0.891	0.875
**AUC**	0.838	0.824	0.821	0.812	0.881	0.854	0.936	0.914
**Sensitivity**	0.782	0.750	0.782	0.792	0.764	0.792	0.891	0.875
**Specificity**	0.812	0.714	0.750	0.714	0.854	0.810	0.875	0.857
**PPV**	0.827	0.750	0.782	0.760	0.857	0.826	0.891	0.875
**NPV**	0.765	0.714	0.750	0.750	0.759	0.773	0.875	0.857

PPV indicates positive prediction value; NPV indicates negative prediction value.

### Construction and Validation of Radiomics Nomogram

**Figure 3:**
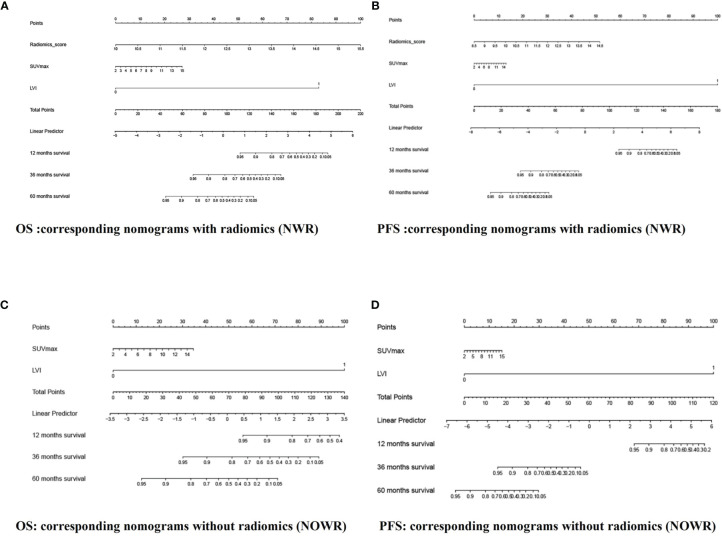
The NWR for OS **(A)** and PFS **(B)** prediction based on rad-score and clinical factors (LVI, SUVmax). The NWOR for OS **(C)** and PFS **(D)** prediction based on clinical factors (LVI, SUVmax).

**Figure 4:**
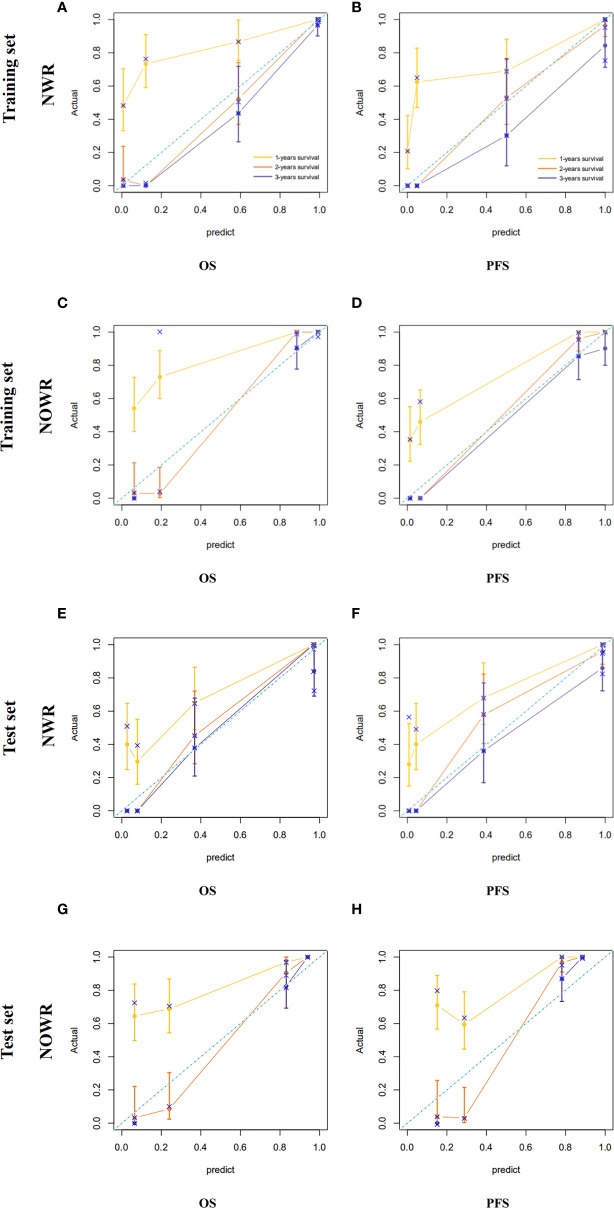
Calibration curve of the NWR for OS **(A)** and PFS **(B)** in the training set. Calibration curve of the NOWR for OS **(C)** and PFS **(D)** in the training set. Calibration curve of the NWR for OS **(E)** and PFS **(F)** in the test set. Calibration curve of the NOWR for OS **(G)** and PFS **(H)** in the test set.

**Figure 5:**
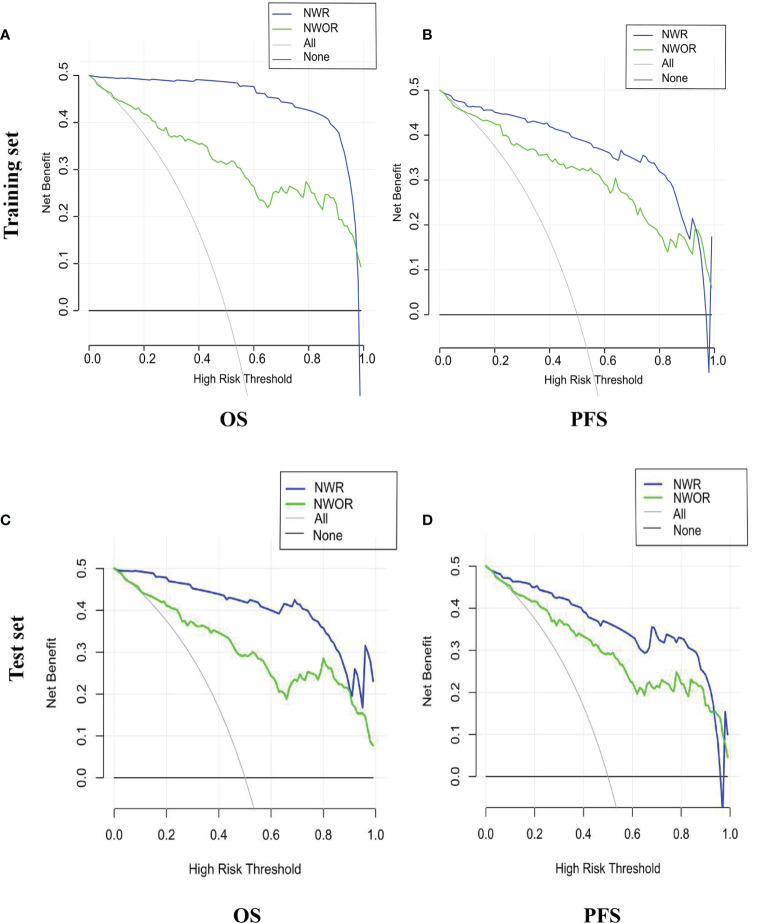
Decision curve of the nomograms for OS **(A)** and PFS **(B)** in the training set. Decision curve of the nomograms for OS **(C)** and PFS **(D)** in the test set.

Among all pathologic and therapeutic factors, SUVmax and pathologic LVI were demonstrated to be associated with survival prognosis, which was incorporated into the nomogram’s construction (**Supplementary Tables 4–7**). Radiomics features for calculating PET/CT Rad-scores of OS and PFS and their importance and significance were displayed in **Tables 3**, **4**. For estimation of OS, the C-index of the NWR in the training set and test set were 0.88 and 0.84, respectively. The C-index of the NWOR in the training set and test set were 0.82 and 0.80, respectively. For estimation of PFS, the C-index of the NWR in the training set and test set were 0.88 and 0.84, respectively. The C-index of the NWOR in the training set and test set were 0.85 and 0.79, respectively. Diagnostic Performance of the NWR and NWOR in **Table 5** and **Figure 3**. The PET/CT-NWR and PET/CT-NOWR, the corresponding calibration curve, and the decision curve were displayed in **Figures 4**, **5**.

**Table 3:** Radiomics features for calculating PET/CT radiomics scores (Rad-scores) of OS and their importance.

Feature name	Importance
original_glszm_SizeZoneNonUniformityNormalized.PET	2.920620505
original_glszm_SmallAreaEmphasis.PET	6.308496129
wavelet.HLH_firstorder_Kurtosis.PET	0.275069109
log.sigma.3.0.mm.3D_ngtdm_Coarseness.PET	1.76E-06
wavelet.HHH_glcm_ClusterShade.PET	10.57007006
wavelet.HLL_glszm_LargeAreaHighGrayLevelEmphasis.PET	9.41E-10
wavelet.LHH_gldm_SmallDependenceEmphasis.CT	4.869217544
wavelet.LHH_glszm_SizeZoneNonUniformityNormalized.CT	-1.937417252
wavelet.LLH_glszm_LargeAreaLowGrayLevelEmphasis.CT	8.70E-06
wavelet.LLL_glcm_Imc1.CT	3.16013583

**Table 4:** Radiomics features for calculating PET/CT radiomics scores (Rad-scores) of PFS and their importance.

Feature name	Importance
log.sigma.3.0.mm.3D_firstorder_90Percentile.PET	9.70279E-05
original_gldm_LargeDependenceLowGrayLevelEmphasis.PET	3.578397243
original_glszm_SmallAreaEmphasis.PET	8.222186126
wavelet.LLH_ngtdm_Contrast.PET	5.71462E-05
log.sigma.3.0.mm.3D_glszm_SmallAreaLowGrayLevelEmphasis.CT	4.247335367
log.sigma.3.0.mm.3D_ngtdm_Coarseness.CT	2.4324E-06
wavelet.HHH_glcm_ClusterShade.CT	8.907641842
wavelet.HLL_glszm_SmallAreaLowGrayLevelEmphasis.CT	4.175090394
wavelet.LHH_glszm_SizeZoneNonUniformityNormalized.PET	-2.014521921
wavelet.LHL_glrlm_GrayLevelNonUniformityNormalized.CT	1.824925527
wavelet.LLL_glcm_Imc1.CT	5.26516043

**Table 5:** Diagnostic Performance of the NWR and NWOR.

Model	OS				PFS			
	Training set		Test set		Training set		Test set	
	c-index	95%CI	c-index	95%CI	c-index	95%CI	c-index	95%CI
NWR	0.88	0.84-0.91	0.84	0.80-0.89	0.88	0.84-0.91	0.84	0.80-0.89
NWOR	0.82	0.77-0.86	0.80	0.75-0.86	0.85	0.81-0.88	0.79	0.73-0.80

### Survival Outcome

As of September 30, 2020, 148 populations had been successfully followed up regarding the OS and PFS. The overall death rate was 50.67% (75/148), and the overall progression rate was 50.67% (75/148). The median OS of all populations was 28.95 months (range, 1-87 months), particularly 16.5 months (range, 1-39 months) for the pathologic LVI-present patients, and 58.7 months (range, 26-87 months) for the pathologic LVI-absent patients. The median PFS of the patients was 17.7 months (range, 1-85 months), particularly 10.4 months (range, 1-26 months) for the pathologic LVI-present patients and 53.3 months (range, 9-85 months) for the pathologic LVI-absent patients. The multivariate Cox regression analysis that SUVmax and pathologic LVI were independent prognostic indicators of both OS [HR=1.210 (95% CI) and 3.814 (95% CI), *P*< 0.001] and PFS [HR=1.233 (95% CI) and 3.988 (95% CI), *P*< 0.001]. Survival curves are displayed in **Supplementary Figures 2–5**.

### Case Study

Two typical cases were chosen by the domain experts—one patient with features predicting LVI-absent status and one with LVI-present status—to illustrate the performance of our model in predicting LVI status and survival outcome. The detailed medical information, including the CT and PET images and fused images for each patient, are shown in **Supplementary Figures 6A, B**. A: Representative PET/CT images in a 60-year-old patient with stage I A gastric cancer, with evidence of LVI-absent status at postsurgical histological analysis after surgery. For predicted LVI-absent patient, OS and PFS were 47.4 and 28.9 months, respectively. B: Representative PET/CT images in a 68-year-old patient with stage II B gastric cancer, with evidence of LVI-present status at postsurgical histological analysis after surgery. For predicted LVI-present patient, OS and PFS were 16.2 and 8.7 months, respectively.

## Discussion

The aim of the present study was to evaluate the diagnostic performance of machine learning models built from a great number of clinicopathological parameters and PET-CT data for predicting pathological LVI status and survival outcomes in GC patients. Our experimental results demonstrated that the PET/CT-RS model incorporating tumor grade and SUVmax exhibited excellent clinical value, which achieved relatively higher AUCs than the PET/CT-RS model did, suggesting the additional value of clinico-pathological variables and metabolic parameters in the identification of LVI status in GC patients. Furthermore, SUVmax and pathologic LVI status were demonstrated to be independent predictors of both OS and PFS, which indicates that SUVmax can serve as a non-invasive bio-marker to facilitate individual treatment strategy schedules.

Previous studies have investigated the potential of baseline metabolic indexes to predict tumor LVI. A previous study conducted by Hyun, SH et al. reported that tumor-to normal liver standardized uptake value ratio (TLR) of the tumor is closely associated with the occurrence of microvascular invasion (MVI) and constructed a predictive model for preoperative prediction of MVI status yielding an AUC of 0.756 (24). In our study, the conventional clinico-pathological indexes (such as age, gender, tumor markers, tumor grade, and so on) and PET metabolic parameters (SUVmax, SUVmean, TLG, and MTV) were analyzed, and only SUVmax and tumor grade were considered as independent LVI predictors, suggesting the traditional parameters extracted from conventional images demonstrate a limited contribution to LVI prediction.

Different from the naked eye discrimination of traditional imaging modality, radiomics analysis enables automatically filtering comprehensive data from images and deeply investigating tumor heterogeneity. In a previous study, the clinical value of radiomics analysis in the prediction of pathological LVI or MVI has been explored. Zhang et al. reported that radiomics models based on magnetic resonance imaging (MRI) and CT could serve as an effective visual prognostic tool for predicting LVI in rectal cancer. It demonstrated the great potential of preoperative prediction to improve treatment decisions (25). Liu et al. explored the use of dynamic contrast-enhanced (DCE)-MRI-based radiomics for preoperative prediction of LVI in invasive breast cancer and found that the DCE-MRI-based radiomics signature in combination with MRI Axillary lymph node (ALN) status was effective in predicting the LVI status of patients with invasive breast cancer before surgery (26). To our best knowledge, we developed the first-of-its-kind machine learning models based on quantitative radiomics signatures derived from preoperative ^18^F-FDG PET/CT images to predict LVI status in GC patients, which may serve as a potential biomarker to supplement the traditional clinical and imaging modalities for personalized treatment in GC patients. Our radiomics models demonstrated favorable predictive efficacy, with high AUCs in the training set and validation set. In the validation set, the prediction accuracy of the integrated model is 0.867, while the accuracy of the PET model and the CT model is 0.756 and 0.733, respectively, which demonstrated that the combined model achieved better predictive efficacy than either the PET-based radiomics signatures or the CT-based radiomics signatures alone. Additionally, with the inclusion of clinical indexes and metabolic parameters in the integrated radiomics model, the predictive performance was improved, suggesting that the clinical factors (tumor grade and lymph node metastasis) and metabolic parameters (SUVmax) played a complementary role in predicting LVI and ultimately contribute to improving the prediction efficacy of the integrated model (training set, validation set AUC are 0.936 and 0.914, respectively).

The current AJCC/UICC guidelines do not include LVI as an independent prognostic indicator of GC in the TNM staging system. However, many studies have shown that LVI is an important prognostic factor for GC after surgical treatment and is associated with tumor recurrence. Patients with LVI had been reported to be associated with poorer prognosis (27–29). Partly in line with previous works, we found that SUVmax and pathological LVI were independent predictors of the survival period, suggesting their clinical usefulness in the long-term management of GC patients. Therefore, in addition to establish a PET/CT-based radiomics signature for the prediction of LVI status, the predictive role of this signature in the survival outcome of GC patients was also explored in this study. Previous research has demonstrated that radiomics analysis can be applied to predict survival outcomes in patients with GC. Jiang et al. analyzed clinico-pathological variables and PET/CT-based radiomics features of 214 GC patients, and a radiomics nomogram with the radiomic signature incorporated was constructed to demonstrate the incremental value of the radiomic signature to the TNM staging system for individualized survival estimation (30).

Different from the previous works, we included LVI and SUVmax as stratifying indexes and explored the survival outcome prediction value of the clinical nomogram. We also provided clinicians an easy-to-use approach to predict survival outcomes by developing a radiomics nomogram that demonstrated favorable discrimination in both the training and testing sets. Additionally, we found an integrated nomogram incorporated PET/CT radiomics and clinical parameters improved survival prediction in GC patients. For estimation of PFS, the c-index of the integrated nomogram is 0.84 in the test set, while the c-index of the clinical parameters-based nomogram is 0.79.

There are some limitations to this study. Firstly, although the final results achieved are ideal, the number of patients included was still limited. A future study with a larger number of samples will be needed to conduct further verification of our results. Secondly, potential selection bias might exist because of the retrospective nature. Therefore, a prospective validation might provide sufficient evidence for clinical application. Thirdly, as tumor segmentation was performed in a manual manner, the exploitation of a more efficient method for tumor segmentation remains an important consideration.

## Conclusion

In summary, this study demonstrated that the application of radiomics analysis based on PET/CT images shows the potential role of preoperative assessment of LVI status. In addition, we developed an easy-to-use tool to predict the survival outcome of patients with GC. Although further investigation, including a much larger number of populations from multicenter, should be carried out to better expand the generalization ability of this method.

## Data Availability Statement

The raw data supporting the conclusions of this article will be made available by the authors, without undue reservation.

## Ethics Statement

The studies involving human participants were reviewed and approved by Harbin Medical University Cancer Hospital. The ethics committee waived the requirement of written informed consent for participation.

## Author Contributions

Conception and design: LY and WC. Collection and assembly of the data: PX. Development of the methodology: ML. Data analysis and interpretation: MW and MP. Manuscript writing: All authors. Manuscript review: LZ and KW. All authors contributed to the article and approved the submitted version.

## Funding

This paper is supported by the National Natural Science Foundation of China General Projects (81571740), Provincial Key Research and Development Program of Heilongjiang Province (GA21C001), Postdoctoral Special Scientific Research Grant of Heilongjiang Provincial Government (LBH-Q17104), Distinguished Young Scientist Funding of Harbin Medical University Affiliated Tumor Hospital (JCQN2019-02), Key Project of the Climbing Funding of the National Cancer Center (NCC201808B019), Haiyan Funding of Harbin Medical University Cancer Hospital (JJQN2019-23). The funders had no role in study design, data collection and analysis, decision to publish, or preparation of the manuscript.

## Conflict of Interest

The authors declare that the research was conducted in the absence of any commercial or financial relationships that could be construed as a potential conflict of interest.

## Publisher’s Note

All claims expressed in this article are solely those of the authors and do not necessarily represent those of their affiliated organizations, or those of the publisher, the editors and the reviewers. Any product that may be evaluated in this article, or claim that may be made by its manufacturer, is not guaranteed or endorsed by the publisher.

## Glossary

**Table:**

LVI	lymph vascular invasion
GC	gastric cancer
2D	two-dimensional
3D	three-dimensional
RSs	radiomics signatures
VOI	volume of interests
Rad-scores	radiomics score
NWR	nomograms with radiomics
NWOR	nomograms without radiomics
SUVmax	maximum standardized uptake value
H&E	hematoxylin and eosin
SUVmean	mean standardized uptake values
^18^F-FDG PET-CT	^18^F-fluorodeoxyglucose positron emission tomography-computed tomography
TLG	total lesion glycolysis
CEA	carcinoembryonic antigen
CA125	carbohydrate antigen 125
CA199	carbohydrate antigen 199
MTV	metabolic tumor volume
ROI	region of interest
ICCs	Intra- and inter-class correlation coefficients
KNN	k-Nearest Neighbor (KNN)
Rad-scores	radiomics score
DCA	Decision curve analysis
ROC	receiver operating curve
AUC	area under the curve
TLR	tumor-to normal liver standardized uptake value ratio
MRI	magnetic resonance imaging
CT	computed tomography
DCE	dynamic contrast-enhanced
ALN	Axillary lymph node
MVI	microvascular invasion
LVSI	lymph vascular space invasion
OS	overall survival
PFS	progression-free survival
DFS	disease-free survival
CECT	contrast-enhanced computed tomography
