# Supplementary material for: Radiomics in Gastric Cancer: First Clinical Investigation to Predict Lymph Vascular Invasion and Survival Outcome Using 18F-FDG PET/CT Images
Source: Front Oncol. 2022 Mar 30;12:836098. doi: 10.3389/fonc.2022.836098 (PMC9005810; doi:10.3389/fonc.2022.836098)

Supplemental Data

Supplementary Material 1 Radiomic Features

This section contains the definitions of the various features that can be extracted using PyRadiomics. They are subdivided into the following classes:

• First Order Statistics (19 features)
• Shape-based (16 features)
• Gray Level Cooccurence Matrix (24 features)
• Gray Level Run Length Matrix (16 features)
• Gray Level Size Zone Matrix (16 features)
• Neigbouring Gray Tone Difference Matrix (5 features)
• Gray Level Dependence Matrix (14 features)

Specific radiomic features are listed as follows:

• First Order Statistics (19 features)

1. Energy
2. Total Energy
3. Entropy
4. Minimum
5. 10th percentile
6. 90th percentile
7. Maximum
8. Mean
9. Median
10. Interquartile Range
11. Range
12. Mean Absolute Deviation (MAD)

13. Robust Mean Absolute Deviation (rMAD)
14. Root Mean Squared (RMS)
15. AbsoluteDeviation
16. Skewness
17. Kurtosis
18. Variance
19. Uniformity

• Shape-based (14 features)

1. Flatness
2. Least Axis Length
3. Major Axis Length
4. Maximum 2D DiameterColumn
5. Maximum 2D DiameterRow
6. Maximum 2D DiameterSlice
7. Maximum 3D Diameter
8. MeshVolume
9. Minor Axis Length
10. Sphericity
11. SurfaceArea
12. Surface Volume Ratio
13. Voxel Volume
14. Elongation

• Gray Level Cooccurence Matrix (24 features)

1. Autocorrelation
2. joint Average
3. Cluster Prominence
4. Cluster Shade

5. Cluster Tendency
6. Contrast
7. Correlation
8. Difference Entropy
9. Difference Variance
10. Difference Average
11. Joint Energy
12. Joint Entropy
13. Informational Measure of Correlation (IMC) 1
14. Informational Measure of Correlation (IMC) 2
15. Inverse Difference Moment (IDM
16. Maximal Correlation Coefficient (MCC
17. Inverse Difference Moment Normalized (IDMN)
18. Inverse Difference (ID)
19. Inverse Difference Normalized (IDN)
20. Inverse Variance
21. Maximum Probability
22. Sum Average
23. Sum Entropy
24. Sum of Squares

- **Gray Level Run Length Matrix (16 features)**

1. Short Run Emphasis (SRE)
2. Long Run Emphasis (LRE)
3. Gray Level Non-Uniformity (GLN)
4. Gray Level Non-Uniformity Normalized (GLNN)
5. Run Length Non-Uniformity (RLN)
6. Run Length Non-Uniformity Normalized (RLNN)
7. Run Percentage (RP)

8. Gray Level Variance (GLV)
9. Run Variance (RV)
10. Run Entropy (RE)
11. Low Gray Level Run Emphasis (LGLRE)
12. High Gray Level Run Emphasis (HGLRE)
13. Short Run Low Gray Level Emphasis (SRLGLE)
14. Short Run High Gray Level Emphasis (SRHGLE)
15. Long Run Low Gray Level Emphasis (LRLGLE)
16. Long Run High Gray Level Emphasis (LRHGLE)

• **Gray Level Size Zone Matrix (16 features)**

1. Small Area Emphasis (SAE)
2. Large Area Emphasis (LAE)
3. Gray Level Non-Uniformity (GLN)
4. Gray Level Non-Uniformity Normalized (GLNN)
5. Size-Zone Non-Uniformity (SZN)
6. Size-Zone Non-Uniformity Normalized (SZNN)
7. Zone Percentage (ZP
8. Gray Level Variance (GLV)
9. Zone Variance (ZV)
10. Zone Entropy (ZE)
11. Low Gray Level Zone Emphasis (LGLZE)
12. High Gray Level Zone Emphasis (HGLZE)
13. Small Area Low Gray Level Emphasis (SALGLE)
14. Small Area High Gray Level Emphasis (SAHGLE)
15. Large Area Low Gray Level Emphasis (LALGLE)
16. Large Area High Gray Level Emphasis (LAHGLE)

• **Neigbouring Gray Tone Difference Matrix (5 features)**

1. Busyness

2. Coarseness
3. Complexity
4. Contrast
5. Strength

• Gray Level Dependence Matrix (14 features)

1. Small Dependence Emphasis (SDE)
2. Large Dependence Emphasis (LDE)
3. Gray Level Non-Uniformity (GLN)
4. Dependence Non-Uniformity (DN)
5. Dependence Non-Uniformity Normalized (DNN)
6. Gray Level Variance (GLV
7. Dependence Variance (DV)
8. Dependence Entropy (DE)
9. Low Gray Level Emphasis (LGLE)
10. High Gray Level Emphasis (HGLE)
11. Small Dependence Low Gray Level Emphasis (SDLGLE)
12. Small Dependence High Gray Level Emphasis (SDHGLE)
13. Large Dependence Low Gray Level Emphasis (LDLGLE)
14. Large Dependence High Gray Level Emphasis (LDHGLE)

Supplementary Table 1 Univariate logistic regression analysis of clinic-pathological factors for predicting LVI status in GC

**Variables**	**β**	**SE**	***P***	**OR (95%CI)**
Gender	-0.2165	0.3606	0.5482	0.805(0.397-1.633)
Age	-0.0111	0.0166	0.5041	0.989(0.957-1.022)
Lymph node metastasis	1.8871	0.4161	<.0001	6.600(2.920-14.919)
Tumor grade	1.9151	0.3535	<.0001	6.787(3.394-13.572)
Molecular subtype	-0.2885	0.1558	0.0640	0.749(0.552-1.017)
T stage	1.2038	0.6703	0.0725	3.333(0.896-12.399)
N stage	1.9126	0.2878	<.0001	6.771(3.852-11.903)
M stage	1.0836	0.5952	0.0687	2.955(0.920-9.490)
cTNM	0.6628	0.4531	0.1435	1.940(0.798-4.716)
CEA	0.2279	0.1939	0.2399	1.256(0.859-1.837)
CA125	0.0034	0.0043	0.4310	1.003(0.995-1.012)
CA199	0.0045	0.0223	0.8390	1.005(0.962-1.049)
SUVmax	0.4340	0.0834	<.0001	1.543(1.311-1.818)
Tumor thickness	0.2626	0.2918	0.3682	1.300(0.734-2.304)
TLG	0.0360	0.0105	<.0001	1.037(1.016-1.058)
SUVmean	0.0520	0.0398	0.1912	1.053(0.974-1.139)
MTV	-0.0123	0.0321	0.7013	0.988(0.928-1.052)

Supplementary Table 2 Multivariate logistic regression analysis of clinic-pathological factors for predicting LVI status in GC

**Variables**	**β**	**SE**	***P***	**OR (95%CI)**
Lymph node metastasis	0.0305	0.6442	0.9622	1.031(0.292-3.644)
Tumor grade	0.3584	0.1012	<.0001	1.431(1.174-1.745)
N	1.2124	0.6887	0.0783	3.362(0.872-12.965)
SUVmax	0.0990	0.0235	<.0001	1.104(1.054-1.156)
TLG	0.00371	0.00415	0.3714	1.004(0.996-1.012)

Supplementary Table 3 Radiomics features and corresponding coefficients for predicting LVI status in GC

Feature name	Corresponding coefficients
['log-sigma-3-0-mm-3D_glszm_GrayLevelNonUniformityNormalized-CT']	9.33527
['log-sigma-2-0-mm-3D_glszm_LargeAreaHighGrayLevelEmphasis-CT']	4.22536
['log-sigma-3-0-mm-3D_firstorder_Kurtosis-CT']	0.00979
['log-sigma-3-0-mm-3D_ngtdm_Busyness-CT']	1.00247
['log-sigma-3-0-mm-3D_glszm_SizeZoneNonUniformityNormalized-PET']	14.03502
['wavelet-HHH_firstorder_Energy-PET']	2.59649
['wavelet-HLH_ngtdm_Coarseness-PET']	0.01455
['wavelet LLH_gldm_LargeDependenceLowGrayLevelEmphasis-PET']	7.33241
['wavelet-LHH_glcm_ClusterShade-PET']	1.45728

The significance of radiomics features is explained as follow:

['log-sigma-3-0-mm-3D_glszm_GrayLevelNonUniformityNormalized-CT']
Gray Level Non-Uniformity Normalized (GLNN)

$$GLNN = \frac{\sum_{i=1}^{N_g}\left(\sum_{j=1}^{N_s}\mathbf{P}(i,j)\right)^2}{N_z^2}$$

GLNN measures the variability of gray-level intensity values in the image, with a lower value indicating agreater similarity in intensity values. This is the normalized version of the GLN formula.

['log-sigma-2-0-mm-3D_glszm_LargeAreaHighGrayLevelEmphasis-CT']
Large Area High Gray Level Emphasis (LAHGLE)

$$LAHGLE = \frac{\sum_{i=1}^{N_g}\sum_{j=1}^{N_s}\mathbf{P}(i,j)i^2j^2}{N_z}$$

LAHGLE measures the proportion in the image of the joint distribution of larger size zones with higher gray-level values.

['log-sigma-3-0-mm-3D_firstorder_Kurtosis-CT']

Kurtosis

$$kurtosis = \frac{\mu_4}{\sigma^4} = \frac{\frac{1}{N_p}\sum_{i=1}^{N_p}(\mathbf{X}(i)-\bar{X})^4}{\left(\frac{1}{N_p}\sum_{i=1}^{N_p}(\mathbf{X}(i)-\bar{X})^2\right)^2}$$

Where μ4 is the 4th central moment.
Kurtosis is a measure of the 'peakedness' of the distribution of values in the image ROI. A higher kurtosis implies that the mass of the distribution is concentrated towards the tail(s) rather than towards the mean.A lower kurtosis implies the reverse: that the mass of the distribution is concentrated towards a spike near the Mean value.

['log-sigma-3-0-mm-3D_ngtdm_Busyness-CT']
Calculate and return the busyness.

$$Busyness = \frac{\sum_{i=1}^{N_g} p_i s_i}{\sum_{i=1}^{N_g}\sum_{j=1}^{N_g}|ip_i - jp_j|}, \text{where } p_i \neq 0, p_j \neq 0$$

A measure of the change from a pixel to its neighbour. A high value for busyness indicates a 'busy' image,with rapid changes of intensity between pixels and its neighbourhood.N.B. if Ng,p = 1, then busyness = $\frac{0}{0}$. If this is the case, 0 is returned, as it concerns a fully homogeneous region.

['log-sigma-3-0-mm-3D_glszm_SizeZoneNonUniformityNormalized-PET']
Size-Zone Non-Uniformity Normalized (SZNN)

$$SZNN = \frac{\sum_{j=1}^{N_s}\left(\sum_{i=1}^{N_g}\mathbf{P}(i,j)\right)^2}{N_z^2}$$

SZNN measures the variability of size zone volumes throughout the image, with a lower value indicating more homogeneity among zone size volumes in the image. This is the normalized version of the SZN formula.

['wavelet-HHH_firstorder_Energy-PET']
Energy

$$energy = \sum_{i=1}^{N_p} (\mathbf{X}(i) + c)^2$$

Here, c is optional value, defined by voxelArrayShift, which shifts the intensities to prevent negative values in X. This ensures that voxels with the lowest gray values contribute the least to Energy, instead of voxels with gray level intensity closest to 0.
Energy is a measure of the magnitude of voxel values in an image. A larger values implies a greater sum of the squares of these values.
['wavelet-HLH_ngtdm_Coarseness-PET']
Coarseness

$$Coarseness = \frac{1}{\sum_{i=1}^{N_g} p_i s_i}$$

Calculate and return the coarseness.
Coarseness is a measure of average difference between the center voxel and its neighbourhood and is an indication of the spatial rate of change. A higher value indicates a lower spatial change rate and a locally more uniform texture.
N.B. $\sum_{i=1}^{Ng}$pisi potentially evaluates to 0 (in case of a completely homogeneous image). If this is the case,an arbitrary value of 106 is returned.
['wavelet LLH_gldm_LargeDependenceLowGrayLevelEmphasis-PET']
Large Dependence Low Gray Level Emphasis (LDLGLE)

$$LDLGLE = \frac{\sum_{i=1}^{N_g} \sum_{j=1}^{N_d} \frac{\mathbf{P}(i,j)j^2}{i^2}}{N_z}$$

Measures the joint distribution of large dependence with lower gray-level values.
['wavelet-LHH_glcm_ClusterShade-PET']
Cluster Shade

$$cluster\ shade = \sum_{i=1}^{N_g} \sum_{j=1}^{N_g} \left(i + j - \mu_x - \mu_y\right)^3 p(i,j)$$

Cluster Shade is a measure of the skewness and uniformity of the GLCM. A higher cluster shade implies greater asymmetry about the mean.

Supplementary Table 4 Univariate logistic regression analysis of clinic- pathological factors for predicting OS in GC

Variables	β	SE	*P*	HR	95%CI
**Gender**	-0.05906	0.25371	0.8159	0.943	0.573-1.550
**Age**	0.01176	0.01177	0.3176	1.012	0.989-1.035
**Lymph node metastasis**	1.58894	0.87531	0.0694	4.8985	0.881-27.236
**Tumor grade**	1.81747	0.99204	0.0669	6.1562	0.881-43.029
**Molecular subtype**	-0.38013	0.31180	0.2227	0.6837	0.371-1.260
**T stage**	1.12827	0.14896	<.0001	3.090	2.308-4.138
**N stage**	1.10053	0.62932	0.0803	3.0058	0.876-10.319
**M stage**	0.75715	0.45129	0.0934	2.1322	0.880-5.164
**cTNM**	0.00191	0.00150	0.2008	1.002	0.999-1.005
**CEA**	0.55896	0.12510	<.0001	1.749	1.369-2.235
**CA125**	0.00130	0.00200	0.5148	1.001	0.997-1.005
**CA199**	0.00174	0.00135	0.1989	1.0017	0.999-1.004
**SUVmax**	0.34318	0.03770	<.0001	1.409	1.309-1.517
**Tumor thickness**	-0.03632	0.19545	0.8526	0.964	0.657-1.414
**TLG**	0.00229	0.00179	0.1996	1.002	0.999-1.006
**SUVmean**	0.04252	0.03134	0.1748	1.0434	0.981-1.110
**MTV**	0.00952	0.02167	0.6605	1.010	0.968-1.053
**LVI**	2.54694	0.36510	<.0001	12.768	6.242-26.115

Note: SUVmax (maximum standardized uptake value); SUV mean (mean standardized uptake value); TLG (total lesion glycolysis); MTV (metabolic tumor volume); CEA (carcinoembryonic antigen); CA125(carbohydrate antigen 125); CA199 (Carbohydrate antigen199); LVI (Lymph vascular invasion).

Supplementary Table 5 Multivariate logistic regression analysis of clinic-pathological factors for predicting OS in GC

Variables	β	SE	*P*	HR	95%CI
**Gender**	-0.32273	0.31504	0.3056	0.724	0.391-1.343
**Age**	0.02401	0.01469	0.1021	1.024	0.995-1.054
**Lymph node metastasis**	-0.26415	0.52031	0.6117	0.768	0.277-2.129
**Tumor grade**	-0.26716	0.45010	0.5528	0.766	0.317-1.850
**Molecular subtype**	-0.26205	0.14256	0.0660	0.769	0.582-1.018
**T stage**	0.62931	0.25947	0.0153	1.876	1.128-3.120
**N stage**	0.28431	0.22227	0.2009	1.329	0.860-2.054
**M stage**	-0.06474	0.69517	0.9258	0.937	0.240-3.661
**cTNM**	0.19943	0.43977	0.6502	1.221	0.516-2.890
**CEA**	0.00411	0.00202	0.0414	1.004	1.000-1.008
**CA125**	0.0008412	0.00295	0.7755	1.001	0.995-1.007
**CA199**	0.0007941	0.0004775	0.0963	1.001	1.000-1.002
**SUVmax**	0.19102	0.05013	0.0001	1.210	1.097-1.335
**Tumor thickness**	0.22283	0.24522	0.3635	1.250	0.773-2.021
**TLG**	-0.0002577	0.00678	0.9697	1.000	0.987-1.013
**SUVmean**	0.10632	0.06524	0.1031	1.112	0.979-1.264
**MTV**	0.02610	0.06845	0.7030	1.026	0.898-1.174
**LVI**	1.33860	0.39832	0.0007	3.814	1.747-8.325

Note: SUVmax (maximum standardized uptake value); SUV mean (mean standardized uptake value); TLG (total lesion glycolysis); MTV (metabolic tumor volume); CEA (carcinoembryonic antigen); CA125(carbohydrate antigen 125); CA199 (Carbohydrate antigen199); LVI (Lymph vascular invasion).

Supplementary Table 6 Univariate logistic regression analysis of clinic- pathological factors for predicting PFS in GC

Variables	β	SE	*P*	HR	95%CI
**Gender**	-0.10469	0.25376	0.6799	0.901	0.548-1.481
**Age**	0.01012	0.01193	0.3964	1.010	0.987-1.034
**Lymph node metastasis**	1.58419	0.87566	0.07043	4.87534	0.876-27.126
**Tumor grade**	1.85311	0.99329	0.06209	6.37963	0.911-44.699
**Molecular subtype**	-0.39995	0.21229	0.05957	0.67035	0.442-1.016
**T stage**	1.19658	0.15391	<.0001	3.309	2.447-4.474
**N stage**	1.12249	0.83004	0.17627	3.07250	0.604-15.633
**M stage**	0.74884	0.45144	0.09716	2.11455	0.873-5.123
**cTNM**	0.00196	0.00151	0.1951	1.002	0.999-1.005
**CEA**	0.55617	0.12497	<.0001	1.744	1.365-2.228
**CA125**	0.00174	0.00199	0.3815	1.002	0.998-1.006
**CA199**	0.00183	0.00135	0.17633	1.00183	0.999-1.004
**SUVmax**	0.36873	0.03961	<.0001	1.446	1.338-1.563
**Tumor thickness**	0.00846	0.19625	0.9656	1.008	0.686-1.482
**TLG**	0.00211	0.00178	0.2356	1.002	0.999-1.006
**SUVmean**	0.04006	0.02093	0.0556	1.041	0.999-1.084
**MTV**	0.00636	0.02161	0.7686	1.006	0.965-1.050
**LVI**	2.59712	0.36679	<.0001	13.425	6.542-27.550

Note: SUVmax (maximum standardized uptake value); SUVmean (mean standardized uptake value); TLG (total lesion glycolysis); MTV (metabolic tumor volume); CEA (carcinoembryonic antigen); CA125(carbohydrate antigen 125); CA199 (Carbohydrate antigen199); LVI (Lymph vascular invasion).

Supplementary Table 7 Multivariate logistic regression analysis of clinic-pathological factors for predicting PFS in GC

Variables	β	SE	*P*	HR	95%CI
**Gender**	-0.60611	0.31560	0.0548	0.545	0.294-1.013
**Age**	0.01928	0.01438	0.1801	1.019	0.991-1.049
**Lymph node metastasis**	-0.40050	0.53071	0.4505	0.670	0.237-1.896
**Tumor grade**	-0.45222	0.45647	0.3218	0.636	0.260-1.557
**Molecular subtype**	-0.33314	0.14079	0.0180	0.717	0.544-0.944
**T stage**	0.77286	0.26415	0.0034	2.166	1.291-3.635
**N stage**	0.38961	0.23180	0.0928	1.476	0.937-2.325
**M stage**	0.37274	0.67376	0.5801	1.452	0.388-5.437
**cTNM**	0.0007087	0.42675	0.9987	1.001	0.434-2.310
**CEA**	0.00461	0.00204	0.0237	1.005	1.001-1.009
**CA125**	0.00341	0.00275	0.2142	1.003	0.998-1.009
**CA199**	0.0007232	0.0004908	0.1406	1.001	1.000-1.002
**SUVmax**	0.20910	0.06159	0.0007	1.233	1.092-1.391
**Tumor thickness**	0.39475	0.25046	0.1150	1.484	0.908-2.425
**TLG**	0.00222	0.00696	0.7502	1.002	0.989-1.016
**SUVmean**	0.08641	0.06586	0.1895	1.090	0.958-1.240
**MTV**	-0.01292	0.06995	0.8535	0.987	0.861-1.132
**LVI**	1.38341	0.41612	0.0008	3.988	1.764-9.016

Note: SUVmax (maximum standardized uptake value); SUVmean (mean standardized uptake value); TLG (total lesion glycolysis); MTV (metabolic tumor volume); CEA (carcinoembryonic antigen); CA125(carbohydrate antigen 125); CA199 (Carbohydrate antigen199); LVI (Lymph vascular invasion).

Supplementary Table 8 Feature importance in NWR for prediction of OS

**Feature name**
original_glszm_SizeZoneNonUniformityNormalized.PET
original_glszm_SmallAreaEmphasis.PET
wavelet.HLH_firstorder_Kurtosis.PET
log.sigma.3.0.mm.3D_ngtdm_Coarseness.PET
wavelet.HHH_glcm_ClusterShade.PET
wavelet.HLL_glszm_LargeAreaHighGrayLevelEmphasis.PET
wavelet.LHH_gldm_SmallDependenceEmphasis.CT
wavelet.LHH_glszm_SizeZoneNonUniformityNormalized.CT
wavelet.LLH_glszm_LargeAreaLowGrayLevelEmphasis.CT
wavelet.LLL_glcm_Imc1.CT

[original_glszm_SizeZoneNonUniformityNormalized.PET]

Size-Zone Non-Uniformity Normalized (SZNN)

$$SZNN = \frac{\sum_{j=1}^{N_s}\left(\sum_{i=1}^{N_g}\mathbf{P}(i,j)\right)^2}{N_z^2}$$

SZNN measures the variability of size zone volumes throughout the image, with a lower value indicating more homogeneity among zone size volumes in the image. This is the normalized version of the SZN formula.

[original_glszm_SmallAreaEmphasis.PET]

SmallAreaEmphasis(SAE)

$$SAE = \frac{\sum_{i=1}^{N_g}\sum_{j=1}^{N_s}\frac{\mathbf{P}(i,j)}{j^2}}{N_z}$$

SAE is a measure of the distribution of small size zones, with a greater value indicative of more smaller size zones and more fine textures.
wavelet.HLH_firstorder_Kurtosis.PET

[log.sigma.3.0.mm.3D_ngtdm_Coarseness.PET]
Coarseness

$$Coarseness = \frac{1}{\sum_{i=1}^{N_g} p_i s_i}$$

Calculate and return the coarseness.
Coarseness is a measure of average difference between the center voxel and its neighbourhood and is an indication of the spatial rate of change. A higher value indicates a lower spatial change rate and a locally more uniform texture.

N.B. $\sum_{i=1}^{Ng}$pisi potentially evaluates to 0 (in case of a completely homogeneous image). If this is the case,an arbitrary value of 106 is returned.

[wavelet.HHH_glcm_ClusterShade.PET]
Cluster Shade

$$cluster\ shade = \sum_{i=1}^{N_g} \sum_{j=1}^{N_g} \left(i + j - \mu_x - \mu_y\right)^3 p(i, j)$$

Cluster Shade is a measure of the skewness and uniformity of the GLCM. A higher cluster shade implies greater asymmetry about the mean.

[wavelet.HLL_glszm_LargeAreaHighGrayLevelEmphasis.PET]
Large Area High Gray Level Emphasis (LAHGLE)

$$LAHGLE = \frac{\sum_{i=1}^{N_g} \sum_{j=1}^{N_s} \mathbf{P}(i, j) i^2 j^2}{N_z}$$

LAHGLE measures the proportion in the image of the joint distribution of larger size zones with higher gray-level values.
[wavelet.LHH_gldm_SmallDependenceEmphasis.CT]
Small Dependence Emphasis (SDE)

$$SDE = \frac{\sum_{i=1}^{N_g} \sum_{j=1}^{N_d} \frac{\mathbf{P}(i,j)}{i^2}}{N_z}$$

A measure of the distribution of small dependencies, with a greater value indicative of smaller dependence and less homogeneous textures.

[wavelet.LHH_glszm_SizeZoneNonUniformityNormalized.CT]
Size-Zone Non-Uniformity Normalized (SZNN)

$$SZNN = \frac{\sum_{j=1}^{N_s}\left(\sum_{i=1}^{N_g}\mathbf{P}(i,j)\right)^2}{N_z^2}$$

SZNN measures the variability of size zone volumes throughout the image, with a lower value indicating more homogeneity among zone size volumes in the image. This is the normalized version of the SZN formula.

[wavelet.LLH_glszm_LargeAreaLowGrayLevelEmphasis.CT]
Large Area Low Gray Level Emphasis (LALGLE)

$$LALGLE = \frac{\sum_{i=1}^{N_g}\sum_{j=1}^{N_s}\frac{\mathbf{P}(i,j)j^2}{i^2}}{N_z}$$

LALGLE measures the proportion in the image of the joint distribution of larger size zones with lower gray-level values.
[wavelet.LLL_glcm_Imc1.CT]
Informational Measure of Correlation (IMC) 1

$$IMC\ 1 = \frac{HXY - HXY1}{\max\{HX, HY\}}$$

IMC1 assesses the correlation between the probability distributions of i and j (quantifying the complexity
of the texture), using mutual information I(x, y):

$$\begin{aligned} I(i,j) &= \sum_{i=1}^{N_g}\sum_{j=1}^{N_g} p(i,j)\log_2\left(\frac{p(i,j)}{p_x(i)p_y(j)}\right) \\ &= \sum_{i=1}^{N_g}\sum_{j=1}^{N_g} p(i,j)\left(\log_2(p(i,j)) - \log_2(p_x(i)p_y(j))\right) \\ &= \sum_{i=1}^{N_g}\sum_{j=1}^{N_g} p(i,j)\log_2\left(p(i,j)\right) - \sum_{i=1}^{N_g}\sum_{j=1}^{N_g} p(i,j)\log_2\left(p_x(i)p_y(j)\right) \\ &= -HXY + HXY1 \end{aligned}$$

However, in this formula, the numerator is defined as HXY - HXY1 (i.e. −I(x, y)), and is therefore ⩽ 0.
This reflects how this feature is defined in the original Haralick paper.
In the case where the distributions are independent, there is no mutual information and the result will therefore be 0. In the case of uniform distribution with complete dependence, mutual information will be equal to log2(Ng).

Finally, HXY − HXY 1 is divided by the maximum of the 2 marginal entropies, where in the latter case of complete dependence (not necessarily uniform; low complexity) it will result in IMC1 = −1, as HX = HY = I(i, j).

Supplementary Table 9 Feature importance in NWR for prediction of PFS

**Feature name**
log.sigma.3.0.mm.3D_firstorder_90Percentile.PET
original_gldm_LargeDependenceLowGrayLevelEmphasis.PET
original_glszm_SmallAreaEmphasis.PET
wavelet.LLH_ngtdm_Contrast.PET
log.sigma.3.0.mm.3D_glszm_SmallAreaLowGrayLevelEmphasis.CT
log.sigma.3.0.mm.3D_ngtdm_Coarseness.CT
wavelet.HHH_glcm_ClusterShade.CT
wavelet.HLL_glszm_SmallAreaLowGrayLevelEmphasis.CT
wavelet.LHH_glszm_SizeZoneNonUniformityNormalized.PET
wavelet.LHL_glrlm_GrayLevelNonUniformityNormalized.CT
wavelet.LLL_glcm_Imc1.CT

[log.sigma.3.0.mm.3D_firstorder_90Percentile.PET]
90th percentile
The 90th percentile of X
[original_gldm_LargeDependenceLowGrayLevelEmphasis.PET]
Large Dependence Low Gray Level Emphasis (LDLGLE)

$$LDLGLE = \frac{\sum_{i=1}^{N_g}\sum_{j=1}^{N_d}\frac{\mathbf{P}(i,j)j^2}{i^2}}{N_z}$$

Measures the joint distribution of large dependence with lower gray-level values

[original_glszm_SmallAreaEmphasis.PET]

SmallAreaEmphasis(SAE)

$$SAE = \frac{\sum_{i=1}^{N_g} \sum_{j=1}^{N_s} \frac{\mathbf{P}(i,j)}{j^2}}{N_z}$$

SAE is a measure of the distribution of small size zones, with a greater value indicative of more smaller size zones and more fine textures.

[wavelet.LLH_ngtdm_Contrast.PET]

Contrast

$$contrast = \sum_{i=1}^{N_g} \sum_{j=1}^{N_g} (i-j)^2 p(i,j)$$

Correlation is a value between 0 (uncorrelated) and 1 (perfectly correlated) showing the linear dependency of gray level values to their respective voxels in the GLCM.

[log.sigma.3.0.mm.3D_glszm_SmallAreaLowGrayLevelEmphasis.CT]

Small Area Low Gray Level Emphasis (SALGLE)

$$SALGLE = \frac{\sum_{i=1}^{N_g} \sum_{j=1}^{N_s} \frac{\mathbf{P}(i,j)}{i^2 j^2}}{N_z}$$

SALGLE measures the proportion in the image of the joint distribution of smaller size zones with lower gray-level values.

[log.sigma.3.0.mm.3D_ngtdm_Coarseness.CT]

Coarseness

$$Coarseness = \frac{1}{\sum_{i=1}^{N_g} p_i s_i}$$

Calculate and return the coarseness.

Coarseness is a measure of average difference between the center voxel and its neighbourhood and is an indication of the spatial rate of change. A higher value indicates a lower spatial change rate and a locally more uniform texture.

N.B. $\sum_{i=1}^{Ng}$pisi potentially evaluates to 0 (in case of a completely homogeneous image). If this is the case,an arbitrary value of 106 is returned.

[wavelet.HHH_glcm_ClusterShade.CT]

Cluster Shade

$$cluster\ shade = \sum_{i=1}^{N_g} \sum_{j=1}^{N_g} \left(i + j - \mu_x - \mu_y\right)^3 p(i,j)$$

Cluster Shade is a measure of the skewness and uniformity of the GLCM. A higher cluster shade implies greater asymmetry about the mean.

[wavelet.HLL_glszm_SmallAreaLowGrayLevelEmphasis.CT]

Small Area Low Gray Level Emphasis (SALGLE)

$$SALGLE = \frac{\sum_{i=1}^{N_g} \sum_{j=1}^{N_s} \frac{\mathbf{P}(i,j)}{i^2 j^2}}{N_z}$$

SALGLE measures the proportion in the image of the joint distribution of smaller size zones with lower gray-level values.

[wavelet.LHH_glszm_SizeZoneNonUniformityNormalized.PET]

Size-Zone Non-Uniformity Normalized (SZNN)

$$SZNN = \frac{\sum_{j=1}^{N_s} \left(\sum_{i=1}^{N_g} \mathbf{P}(i,j)\right)^2}{N_z^2}$$

SZNN measures the variability of size zone volumes throughout the image, with a lower value indicating more homogeneity among zone size volumes in the image. This is the normalized version of the SZN formula.

[wavelet.LHL_glrlm_GrayLevelNonUniformityNormalized.CT]

Gray Level Non-Uniformity Normalized (GLNN)

$$GLNN = \frac{\sum_{i=1}^{N_g} \left(\sum_{j=1}^{N_s} \mathbf{P}(i,j)\right)^2}{N_z^2}$$

GLNN measures the variability of gray-level intensity values in the image, with a lower value indicating a greater similarity in intensity values. This is the normalized version of the GLN formula.

[wavelet.LLL_glcm_Imc1.CT]

Informational Measure of Correlation (IMC) 1

$$IMC\ 1 = \frac{HXY - HXY1}{\max\{HX, HY\}}$$

IMC1 assesses the correlation between the probability distributions of i and j (quantifying the complexityof the texture), using mutual information I(x, y):

$$I(i,j) = \sum_{i=1}^{N_g}\sum_{j=1}^{N_g} p(i,j)\log_2\left(\frac{p(i,j)}{p_x(i)p_y(j)}\right)$$

$$= \sum_{i=1}^{N_g}\sum_{j=1}^{N_g} p(i,j)\big(\log_2(p(i,j)) - \log_2(p_x(i)p_y(j))\big)$$

$$= \sum_{i=1}^{N_g}\sum_{j=1}^{N_g} p(i,j)\log_2\big(p(i,j)\big) - \sum_{i=1}^{N_g}\sum_{j=1}^{N_g} p(i,j)\log_2\big(p_x(i)p_y(j)\big)$$

$$= -HXY + HXY1$$

However, in this formula, the numerator is defined as HXY - HXY1 (i.e. −I(x, y)), and is therefore ⩽ 0.
This reflects how this feature is defined in the original Haralick paper.
In the case where the distributions are independent, there is no mutual information and the result will therefore be 0. In the case of uniform distribution with complete dependence, mutual information will be equal to log2(Ng).
Finally, HXY − HXY 1 is divided by the maximum of the 2 marginal entropies, where in the latter case of complete dependence (not necessarily uniform; low complexity) it will result in IMC1 = −1, as HX = HY = I(i, j).

Supplementary Figure 1 Flow chart

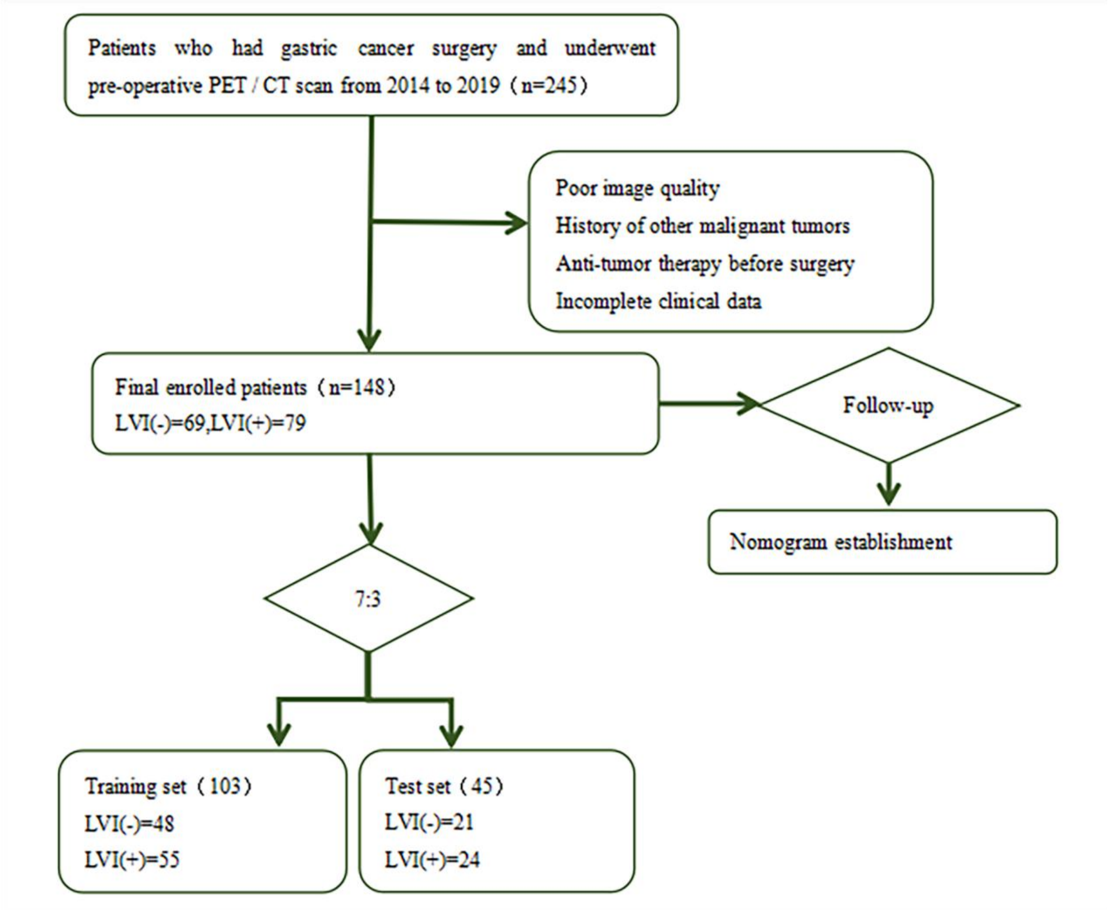

Supplementary Figure 2 Overall survival (OS) curves according to pathologic LVI status

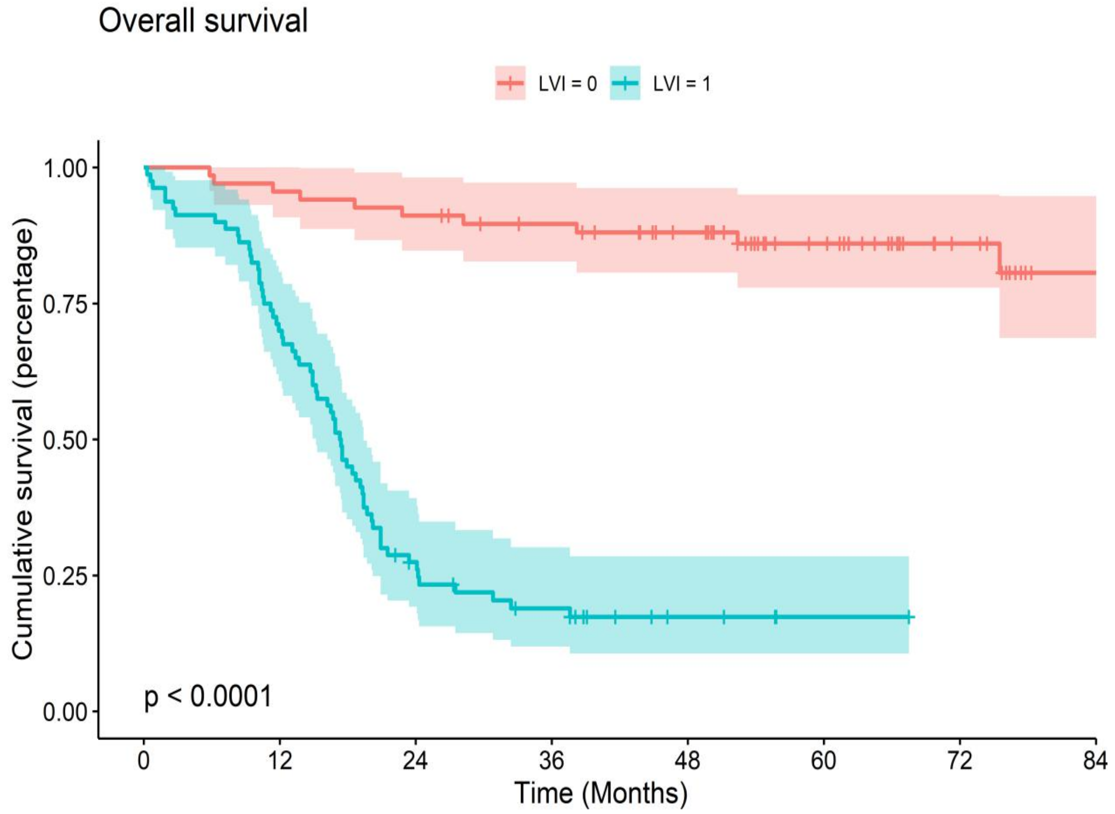

Supplementary Figure 3 Progression-free survival (PFS) curves according to pathologic LVI status

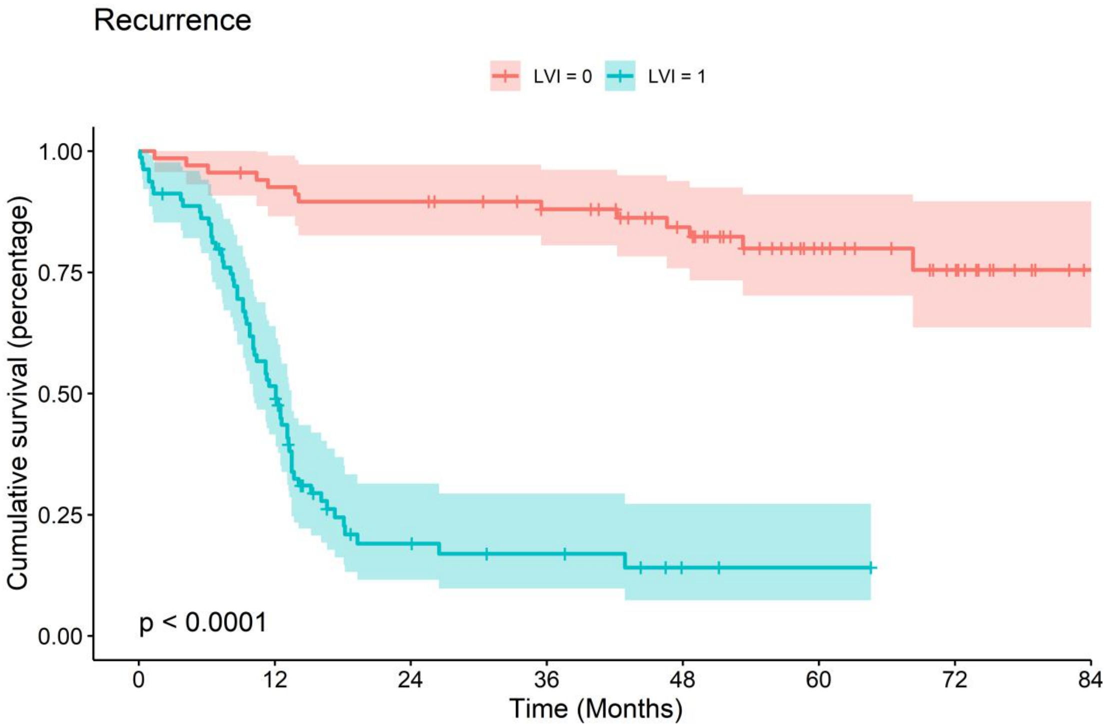

Supplementary Figure 4 Overall survival (OS) curves according to maximum standardized uptake values (SUVmax) values

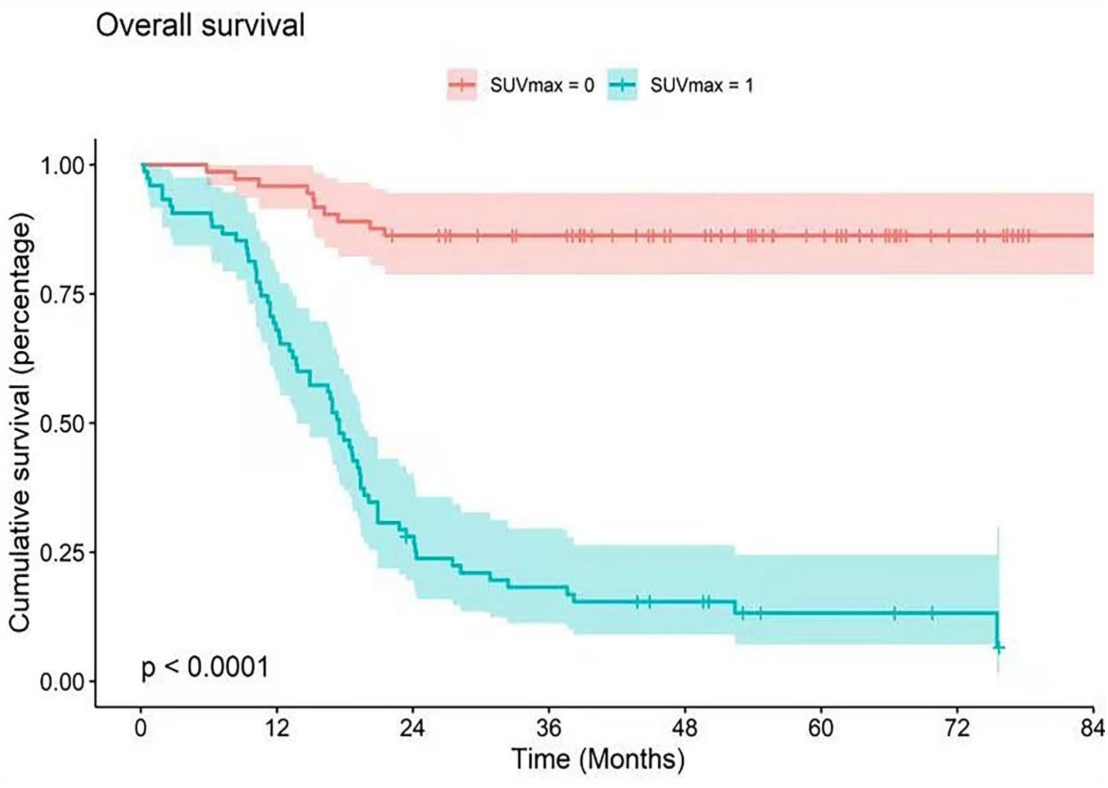

Supplementary Figure 5 Progression-free survival (PFS) curves according to maximum standardized uptake values (SUVmax) values

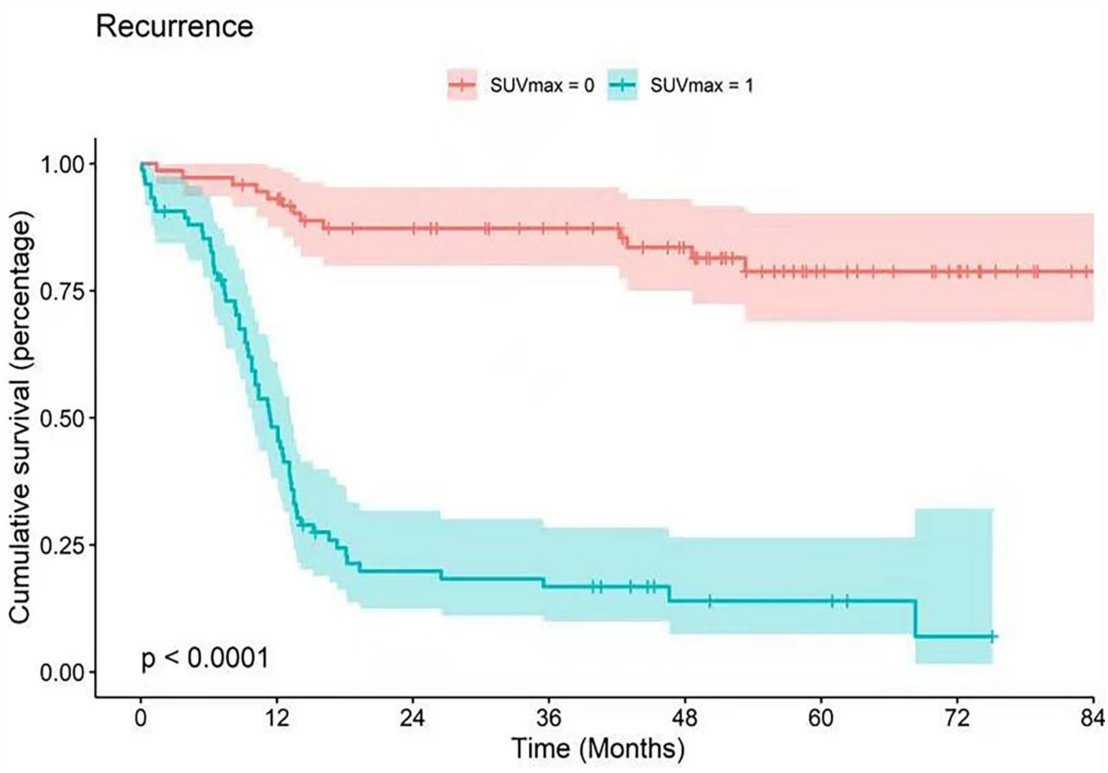

Supplementary Figure 6 Representative PET/CT images in one patient with features predicting LVI-absent status and one with LVI-present status

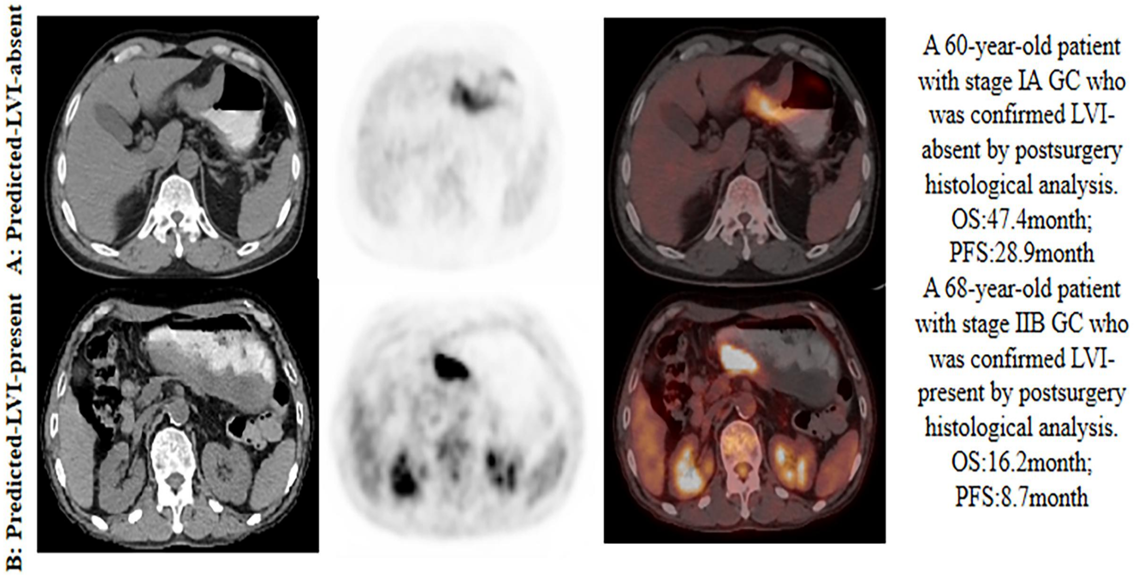

Supplement: Supplementary file 1 [file Presentation_1.pdf]
